# Inversion of the Uterus

**Published:** 1868-02

**Authors:** 

**Affiliations:** Altkirch


### INVERSION OF THE UTERUS.—NEW MODE OF REDUCTION.

By Dr. FESSENMAYER, of Altkirch.

The 29th December, 1863, I was called to a young woman, 20 years old, primaparous, delivered on that day, and subsequently having flooded considerably. I recognized by the touch a complete inversion of the uterus. The os tincæ, which bounded the summit of the vaginal tumor, was very close. Attempts at reduction were fruitless, and renewed the hemorrhage. Moreover, the volume of the uterus was too great for reduction through the os tincæ. I prescribed hemostatics and astringent injections.

A few days before, I had made use of an India-rubber ball as a tampon, for a case of partial adherence of the placenta to the cervix uteri. Labor commenced as usual, with a very abundant flow of blood. The inflated ball arrested the hemorrhage, at the same time promoting the contractions. The dilated neck allowed the introduction of the hand to detach the placenta and disengage the head, which was presenting. The numerous advantages offered in obstetrical practice by the inflated ball presented themselves to my mind, on returning home from my visit to this lady. I observed, also, that her perineum and fourchette were intact, and immediately conceived the idea of employing the rubber ball to attempt the reduction of the inverted uterus. I proposed this operation to the lady, making a delay of its trial for several weeks, that the uterus might become disgorged. It was six months before I had an opportunity to put in practice this plan of operation. The existence of uterine inversion was unknown to the midwife and the other wise heads of the neighborhood. I myself had only lived there six months, and public opinion was not favorable to me.

July 10th, 1863, she was brought to me in a carriage. Her features were greatly changed; she was very anæmic; her lower extremities were œdematous; a chronic bronchitis was wearing her out. From the vagina flowed a grayish sanies, profuse and fetid; her catamenia had scarcely an eight days' interval between periods. She was too weak to sit up, and they had to bring her from the carriage on a bed. On examination by the touch, I found the uterus inverted, very small, quite disgorged; the os tincæ was hard and flattened upon the neck.

As this woman lived several miles from my residence, I had to give my instructions to her father, and explained to him the results which I expected from the inflation of the tampon ball. The one I had was tolerably strong, and one could easily give it the volume of a child's head at the eighth month of intra-uterine life. I required the woman to bear a pressure slightly increased every day, so as to attain, at the end of several days, the above-mentioned volume. I prescribed emollient injections morning and evening, and advised her to remove the apparatus at stool and in urinating, if there was too much pressure.

On the morning of the ninth day, the father used rather more force than usual in distending the ball, and she suffered all day from colic and pains extending from the hypogastrium to the breast. She had the fortitude not to alter the apparatus, thinking that it was producing its effects, and her instincts did not fail her. About 4 P.M., the pains ceased, and the next day the father no longer found the tumor in the vagina. The introduction of the ball was, however, continued.

Three days after, 22d July, I visited my patient, who considered herself cured. The vaginal discharge was slight. By the touch I found the uterus in place; the finger still easily penetrated the neck, in the midst of which was distinctly felt a transverse ring, the size of the little finger. A fortnight subsequently, this little opening was still perceptible in the anterior and middle part of the neck, which besides was almost firm.

From the 20th July she began to mend. The catamenia appeared three weeks later, thenceforth always regular as before her marriage. The cough slowly abated. Pills of protiodide of iron contributed to the recovery, and the anæmia rapidly disappeared.

This new method of reducing an inverted uterus had sustained the proof of trial, and given success in a case where I dared not hope for it. But how would it answer in a recent case of inversion? The following observation allows me to state fortunately.

The 4th December, 1865, I was called to a lady delivered two days before. There had been profuse flooding. She had vomiting and a swollen abdomen. I found her very weak and anæmic. The abdomen was distended. In the hypogastrium there was a large tumor, which I took to be the bladder, from its elasticity. In endeavoring to confirm my diagnosis by vaginal examination, I was astonished to find a complete inversion of the uterus. I drew off, with the catheter, four *litres* of urine; I treated constipation with enemata and a laxative; and postponed for several weeks attempts at reduction, by reason of the large volume of the uterus. One month later, I sent to her husband, not a tampon ball, for her perineum was impaired, but a large India-rubber pessary, with an inflater. This pessary presented a flat, horizontal portion, which gives a larger point of support when the perineum is ruptured.

After applying the apparatus and indicating the method of distending the pessary, I required the husband to act as in the preceding case. At the time treatment was begun, the uterus was engorged, but its volume was little greater than normal. She was always œdomatous, on account of excessive anæmia. Notwithstanding this unfavorable condition, toward the evening of the third day, as I had foreseen, she felt *strong pains extend from the lower abdomen toward the breast.* At the end of four hours they ceased, and the uterus was reduced.

The catamenia returned only on the 1st March. The 10th March, 1866, I could assure myself that the uterus was in its natural place and had recovered its normal volume. This woman was not confined to bed during treatment, as she was obliged to attend to household duties. An abundant serous discharge existed from delivery until the reduction of the uterus; from this time it entirely disappeared. She rapidly recovered, and her health soon became perfect.—*Gazette Medicale de Strasbourg.*

